# The influence of selective serotonin reuptake inhibitors on stroke outcome: a systematic review and meta-analysis

**DOI:** 10.3389/fneur.2026.1894976

**Published:** 2026-08-26

**Authors:** Yuanhui Sun, Siyu Mu

**Affiliations:** 1 Department of Pediatric, The First Affiliation Hospital of China Medical University, Shenyang, China; 2 Department of Neurology, The First Hospital of China Medical University, Shenyang, China

**Keywords:** meta-analysis, prognosis, selective serotonin reuptake inhibitor, SSRI, stroke, systematic review

## Abstract

**Objective:**

Selective serotonin reuptake inhibitors (SSRIs) are recognized for their antidepressant effects and their potential role in promoting neuroplasticity. This systematic review and meta-analysis aimed to evaluate whether SSRIs improve prognostic outcomes in patients with stroke compared with placebo or no intervention.

**Methods:**

We systematically searched the Cochrane Library, PubMed, and Embase databases to identify randomized controlled trials comparing SSRIs with placebo or no intervention in patients with stroke. Continuous outcomes were pooled using weighted mean differences (WMDs), and dichotomous outcomes were pooled using risk ratios (RRs), both with 95% confidence intervals (CIs). Fixed-effect or random-effects models were selected according to the degree of statistical heterogeneity for each outcome.

**Results:**

Eleven reports from nine unique randomized controlled trials, involving 7,604 unique participants, were included. In exploratory analyses stratified by follow-up duration, SSRI use at 3 months was associated with better modified Rankin Scale (mRS) responder status and a greater absolute reduction in National Institutes of Health Stroke Scale score (absNIHSS) (RR 2.46, 95% CI 1.74–3.48; WMD 0.57, 95% CI 0.11–1.03). However, the 3-month mRS finding was derived from a single trial and should therefore be interpreted with particular caution. At 6 months, SSRI use was associated with a smaller absolute reduction in NIHSS score (WMD −0.23, 95% CI–0.45 to −0.02), but this finding should also be interpreted cautiously because the absNIHSS analysis showed substantial heterogeneity. At 12 months, SSRI use was associated with a lower risk of recurrent stroke (RR 0.65, 95% CI 0.47–0.89), particularly recurrent ischemic stroke (RR 0.57, 95% CI 0.40–0.80). These findings suggest that the observed associations between SSRI use and stroke outcomes varied across follow-up-duration subgroups. However, these subgroup patterns should be interpreted cautiously because they may reflect differences in study populations, follow-up designs, attrition, rehabilitation strategies, and trial weighting rather than a confirmed biological time-dependent effect. No statistically significant differences were observed in thrombotic events, bleeding events, mortality, cardiac adverse events, or drug-specific subgroup analyses.

**Conclusion:**

SSRI use after stroke may be associated with selected exploratory short-term functional signals and a lower long-term risk of recurrent stroke, particularly recurrent ischemic stroke. However, the apparent short-term mRS benefit was driven by a single trial and should not be interpreted as a robust or generalizable clinical effect. The mid-term absNIHSS findings did not support a sustained functional benefit and were accompanied by substantial heterogeneity. Therefore, the functional outcome findings should be interpreted as exploratory rather than definitive. The observed associations should be interpreted cautiously because they were derived from exploratory follow-up-duration-stratified analyses and represent subgroup-level associations rather than evidence of a causal temporal or biological effect of SSRIs. Further well-designed randomized controlled trials are needed to clarify the optimal timing, duration, and safety of SSRI therapy after stroke.

## 1 Introduction

Stroke is the second leading cause of mortality worldwide and the third leading cause of combined death and disability (1). Current stroke care involves a continuum of management, including acute treatment, post-acute rehabilitation, and long-term secondary prevention. In the acute phase, eligible patients may receive reperfusion therapies, such as intravenous thrombolysis and, when indicated, endovascular thrombectomy, together with stroke-unit care, antithrombotic therapy, complication management, and early secondary prevention (2, 3). After the acute phase, multidisciplinary rehabilitation, including physical therapy, occupational therapy, speech and language therapy, and long-term functional support, plays an essential role in promoting functional recovery and reducing disability (2, 3). However, despite advances in acute stroke treatment and rehabilitation, many patients continue to experience persistent neurological deficits. Therefore, substantial research has focused on pharmacological agents and therapeutic strategies that may enhance neuroplasticity and improve post-stroke prognosis. Presently, the use of SSRIs has garnered considerable attention in this context.

Selective serotonin reuptake inhibitors (SSRIs) have been widely used as first-line pharmacological treatments for major depression for many years (4). Recently, several clinical studies have suggested that SSRIs may improve clinical recovery after stroke independently of their antidepressant effects by promoting neuroplasticity and enhancing motor recovery (5, 6). Among these agents, fluoxetine has been most extensively investigated and has been reported to exert neuroprotective and motor-modulating effects in previous studies (7, 8). Typically, a daily dose of 20 mg has been used in most previous trials involving SSRIs in stroke, with adjustments made according to patients’ clinical conditions (9).

Previous Cochrane Reviews of SSRIs for Stroke Recovery published in 2019 and 2021 suggested that SSRIs may improve some recovery outcomes in patients with stroke, while also being associated with several adverse effects (9, 10). However, whether SSRIs provide clinically meaningful prognostic benefits in these patients, and which factors may influence such effects, remains uncertain. Several studies have shown that early treatment with SSRIs may improve neurological functional prognosis in patients with ischemic stroke (11, 12). Conversely, some randomized controlled trials (RCTs) have shown no delayed or sustained benefits on functional outcomes (13, 14). Therefore, existing evidence has not reached a consistent conclusion on this issue.

Given the conflicting results regarding the prognostic outcomes of SSRIs in patients with stroke, we conducted a systematic review and meta-analysis of RCTs to examine the effects of SSRIs compared with placebo or no intervention on prognostic outcomes. This review assessed various scale-based functional outcomes and clinical events in patients with stroke. Furthermore, we stratified the outcomes according to follow-up duration to explore potential differences across short-, mid-, and long-term follow-up periods.

## 2 Methods

This systematic review was conducted and reported in accordance with the Preferred Reporting Items for Systematic Reviews and Meta-Analyses 2020 statement (PRISMA 2020) (15). The review protocol was registered in the International Prospective Register of Systematic Reviews (PROSPERO; registration number: CRD42022349722).

### 2.1 Information sources and search strategy

We searched the Cochrane Library, PubMed, and Embase from database inception to 7 July 2022 using free-text terms and controlled vocabulary. To update the review, we repeated the search using the same search strategy and eligibility criteria for the period from 1 July 26, 2026 2022 to 7 July 2026. The final searches were run on 7 July 2026. No additional eligible randomized controlled trials were identified. The complete database-specific search strategies and exact search dates are provided in Supplementary Table S1.

### 2.2 Eligibility criteria

**Figure 1:**
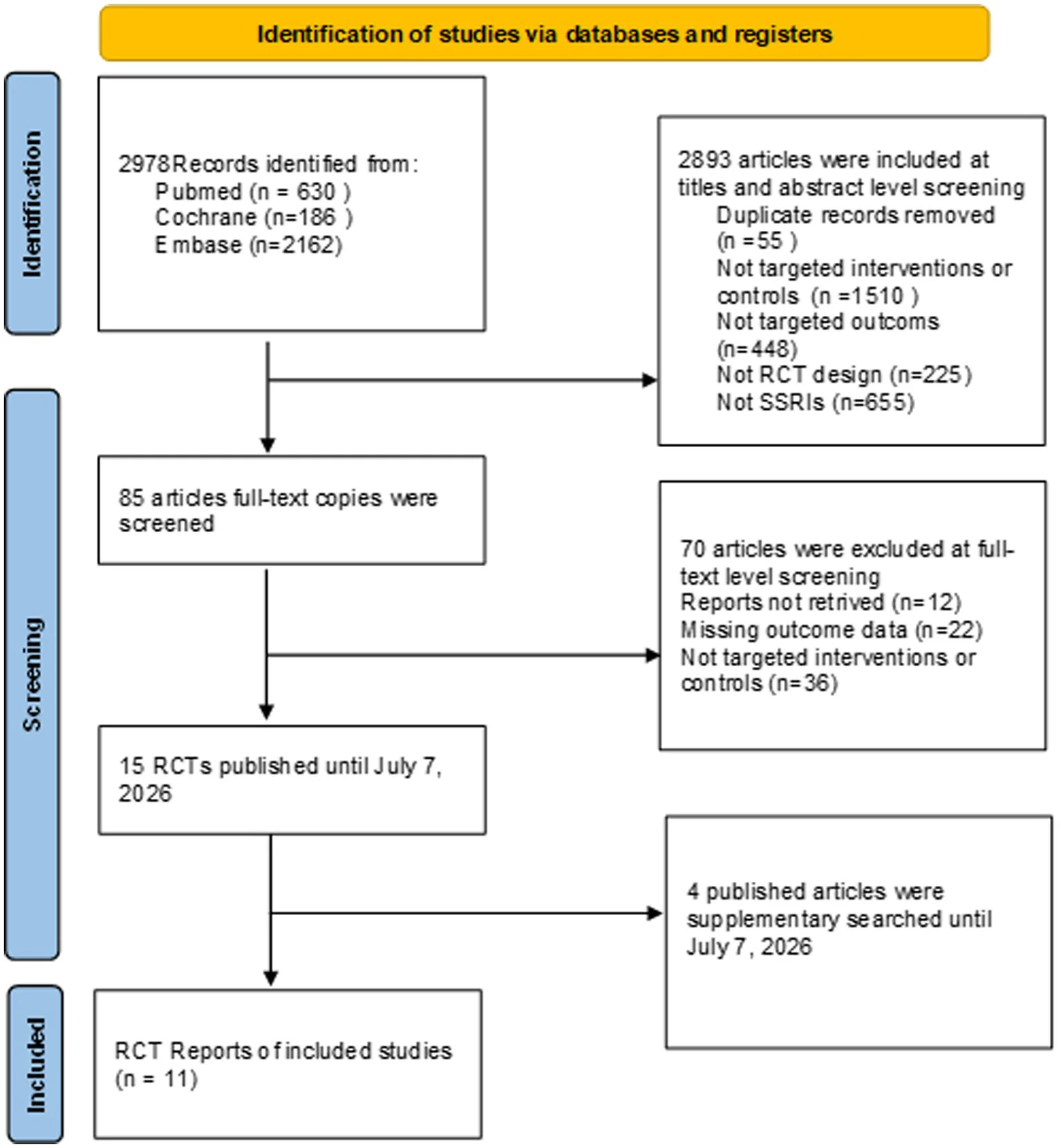
PRISMA flow chart of the identification and selection of relevant studies (database search).

We included RCTs that compared the impact of SSRIs versus placebo or no intervention. The definition of SSRIs used is provided in Supplementary Data S1. Details regarding the route and dosage of drug administration can be found in Figure 1. The target population for these trials consisted of patients diagnosed with ischemic stroke through methods such as CT or MRI or by a physician’s diagnosis. The intervention involved the use of SSRIs, which encompassed fluoxetine, citalopram, and escitalopram, while the comparator group received usual care, placebos, or conventional therapy. The primary outcomes assessed in our systematic review were recurrent stroke, mRS (modified Rankin Scale), and the absolute decrease in the National Institute of Health Stroke Scale (absNIHSS). Secondary outcomes included bleeding events, cardiac adverse events, death events, thrombotic events, and drug-specific subgroup analyses. We did not impose any restrictions on the length of follow-up or the duration of treatment. Additional interventions were allowed for all patients in both arms as long as the only difference between the groups was the use of an SSRI.

### 2.3 Study selection, data collection

Two reviewers (SM and YS) independently screened the titles and abstracts of articles obtained from both the original and updated searches to determine their suitability for full-text review. Subsequently, the full texts of these articles were thoroughly examined to assess their eligibility for inclusion in the systematic review. For records without immediately available full text, two reviewers attempted retrieval through institutional access, publisher websites, DOI-based searches, Google Scholar, database-linked full-text services, trial registries, interlibrary loan pathways, and author contact when feasible. Records that remained unavailable were classified separately as “full text unavailable” and were excluded from quantitative synthesis because eligibility, intervention details, outcome definitions, risk of bias, and extractable data could not be reliably verified. Any uncertainties or disagreements that arose were addressed through consensus discussions. Detailed records were maintained regarding the reasons for excluding articles during the full-text screening stage. All studies that explicitly reported any of the relevant outcomes were included in the qualitative analysis. The characteristics and findings of the study were independently extracted into a standardized spreadsheet, which included the main characteristics of the sample, intervention, comparison, and outcome. All decisions regarding data handling and any assumptions made were reached through consensus, and these decisions were established prior to conducting any statistical analyses. Multiple publications originating from the same randomized trial were linked and treated as reports of a single unique RCT. For each outcome and follow-up time point, data were extracted from the most complete and relevant report. When different reports presented outcomes at different follow-up times, the corresponding report was used for that specific time point. No participant cohort was included more than once within any individual meta-analysis.

### 2.4 Risk of bias assessment

Two authors, SM and YS, independently assessed the risk of bias in the included randomized controlled trials using Review Manager version 5.4. Risk of bias was assessed using the traditional Cochrane Risk of Bias tool. This framework was retained because RoB 1 had been prespecified and applied during the original conduct of this review, and the same assessment approach was used consistently for all included trials during both the original and updated review process. Although the updated Cochrane RoB 2 tool provides a more structured domain-based assessment for randomized trials, replacing RoB 1 with RoB 2 at the revision stage could introduce *post hoc* changes in judgment criteria and reduce consistency with the original protocol and analyses. Therefore, RoB 1 was retained for the main risk-of-bias assessment. The assessed domains included random sequence generation, allocation concealment, blinding of participants and personnel, blinding of outcome assessment, incomplete outcome data, selective reporting, and other potential sources of bias. Studies were categorized overall as having low, unclear, or high risk of bias. The potential influence of trials judged to be at high risk of bias was considered in the interpretation of pooled estimates, particularly for outcomes to which these trials contributed data. The risk-of-bias assessment plots are displayed in Supplementary Figure S1.

### 2.5 Statistical analysis and synthesis of results

For our statistical analysis and the creation of forest plots, we employed the Review Manager (version 5.4). We reported pooled dichotomous data using risk ratios (RRs), reporting 95% CIs, and corresponding *p* values. Additionally, we reported pooled continuous variables using weighted mean difference (WMD), reporting 95% CIs, and corresponding *p* values. Heterogeneity was evaluated using the I2 test. When *I*^2^ exceeded 50% with a corresponding heterogeneity test *p* value <0.05, heterogeneity was considered substantial, and a random-effects model was used. When heterogeneity was not substantial, a fixed-effect model was used. To improve transparency and reproducibility, the model used for each major outcome was explicitly reported in the corresponding Results section. Effect measures were reported consistently, with RRs used for dichotomous outcomes and WMDs used for continuous outcomes. For results that exhibited significant heterogeneity, we performed sensitivity analyses by systematically omitting one study at a time to assess the impact on the overall findings. In addition, for outcomes including trials judged to be at high risk of bias, we considered whether these studies could have influenced the direction, magnitude, or certainty of the pooled estimates. Because only one trial judged to be at high risk of bias contributed to each affected outcome domain, and because exclusion of these trials would leave only one study or a very small number of studies in some follow-up-duration subgroups, a separate quantitative sensitivity analysis excluding high-risk trials was not considered sufficiently stable or informative. The potential influence of these trials was therefore assessed qualitatively by considering the affected outcomes, direction and magnitude of the pooled estimates, statistical heterogeneity, number of contributing studies, and risk-of-bias domains. Findings involving high-risk trials were interpreted cautiously and regarded as having reduced certainty. When a subgroup result was based on only one study, we did not interpret it as a pooled effect. Instead, it was described as a single-study finding and was considered hypothesis-generating. For outcomes with substantial heterogeneity, particularly mRS and absNIHSS, clinical interpretation was made cautiously by considering study-level differences, sample size, follow-up duration, outcome definitions, and the influence of individual trials. Potential sources of heterogeneity were further explored qualitatively, including differences in SSRI type and dosage, timing and duration of SSRI administration, baseline stroke severity, trial design, rehabilitation strategies, outcome definitions, follow-up schedules, risk of bias, and the influence of large multicenter randomized controlled trials on pooled estimates. Additionally, we conducted subgroup analyses based on the duration of follow-up. These subgroup analyses were exploratory and were performed to describe whether pooled estimates differed across follow-up categories. They were not designed to establish a causal time-dependent effect of SSRIs on stroke outcomes. Therefore, subgroup differences were interpreted as observed associations only. Alternative explanations, including differences in study populations, follow-up duration and assessment schedules, missing data or attrition over time, rehabilitation strategies, trial design, outcome definitions, and the disproportionate influence of large multicenter RCTs, were considered when interpreting the results. To investigate potential publication bias, we employed funnel plots when pooling results from more than 10 trials.

## 3 Results

### 3.1 Included studies

The initial search yielded a total of 2,978 records, of which 2,923 remained after removing duplicates. Following a thorough screening process involving the evaluation of titles and abstracts, 2,838 records were excluded. During the subsequent full-text screening, an additional 70 records were excluded. After duplicate removal, title and abstract screening, and full-text assessment, no additional eligible randomized controlled trials were identified. Therefore, the total number of included studies remained unchanged. Ultimately, 11 reports corresponding to 9 unique RCTs were included. These trials comprised 7,604 unique randomized participants. The 12-month AFFINITY and EFFECTS publications were follow-up reports of their respective parent RCTs and were not counted as additional independent trials or participant cohorts. Multiple reports from the same trial were linked during data extraction, and no participant cohort was included more than once within any individual meta-analysis (14, 16–25) were included for qualitative analysis. The flow chart is displayed in Figure 1. The PRISMA flow diagram was updated to include the supplementary search conducted from 1 July 2022 to 7 July 2026. The sample sizes ranged from 61 to 3,127 patients, with a total of 7,604 patients included in our review. To explore whether the pooled estimates differed according to follow-up duration, we categorized the included studies into three groups: 3 months, 6 months, and equal to or exceeding 12 months. Because some follow-up subgroups contained only one trial, these subgroup findings were interpreted descriptively rather than as robust pooled estimates. These analyses were interpreted as exploratory follow-up-duration-stratified analyses rather than as evidence of a causal time-dependent effect. Because trials differed in study population, follow-up design, treatment duration, rehabilitation background, and sample size, comparisons across follow-up subgroups were considered descriptive rather than confirmatory. The main characteristics and the relevant results extracted from the included studies are presented in Table 1 and Supplementary Table S2. The sample sizes ranged from 61 to 3,127 patients, with a total of 7,604 patients included in our review. Because the updated search did not identify additional eligible randomized controlled trials, the study number, total sample size, and pooled estimates remained unchanged. The average age ranged from 61.53 to 71.35 years old, and around 62.89% were males. The study publication dates varied from 2008 to 2020, with follow-up analyses published in 2021. All of the studies involved stroke patients, with 8 of them focusing on fluoxetine, 2 on citalopram, and 1 on escitalopram. Notably, the three largest studies accounted for 77.68% of the total patient population [EFFECTS (24), AFFINITY (23), and FOCUS (21)]. This distribution suggests that the pooled estimates may have been influenced disproportionately by large multicenter fluoxetine trials, whereas evidence for other SSRIs was comparatively limited. Differences in SSRI type, trial size, treatment duration, and follow-up design were therefore considered important potential sources of clinical heterogeneity.

**Table 1:** Main characteristics of included studies.

Study	Unique trial/ Trial family	Report type	Publication date	Location	Study type	Study size	Age (mean, SD)	Gender (female, male)	Baseline NIHSS (mean, SD)	Baseline mRS of intervention	Baseline mRS of placebo	Route and dose of drug intake	Follow-up time	Intervention	Relavent outcomes
Oskouie et al. (18)	Oskouie et al. trial	Primary 3-month report	2017	Iran	RCT	123	66.39, 11.09	60, 63	10.6, 4.73	Favorable score (mRS = 0–2) :466 (63%) Moderate disability (mRS = 3):168 (23%) Moderate to severe disability (mRS = 4): 46(6%) Severe disability (mRS = 5): 23 (37.10%)	Favorable score (mRS = 0–2): 475 (64%) Moderate disability (mRS = 3):164 (22%) Moderate to severe disability (mRS = 4): 48 (7%) Severe disability (mRS = 5): 34 (55.74%)	oral; 20 mg once daily	3 months	Citalopram	Mortality, mRS, NIHSS, recurrent stroke, cardiac adverse event
Lundström et al. (24)	EFFECTS	Primary 6-month report	2020	Sweden	RCT	1,500	70.8, 10.9	575, 925	3.7, 2.97	Favorable score (mRS = 0–2): 0 Moderate disability (mRS = 3):18 (29.03%) Moderate to severe disability (mRS = 4): 21 (33.87%) Severe disability (mRS = 5): 23 (37.10%)	Favorable score (mRS = 0–2) :0 Moderate disability (mRS = 3):18 (29.03%) Moderate to severe disability (mRS = 4): 21 (33.87%) Severe disability (mRS = 5): 23 (37.10%)	oral; 20 mg once daily	6 months	Fluoxetine	Mortality, mRS, NIHSS, recurrent stroke, cardiac adverse event, thrombotic event, bleeding event
Lundström et al. (23)	EFFECTS	12-month follow-up report	2021	Sweden	RCT	1, 500	70.8, 10.9	575, 925	3.7, 2.97			oral; 20 mg once daily	12 months	Fluoxetine	Mortality, mRS
Hankey et al. (14)	AFFINITY	Primary 6-month report	2020	Australia, New Zealand, Vietnam	RCT	1, 280	64.05, 12.36	476, 804	6, 0.97	avorable score (mRS = 0–2):432 (69%) Moderate disability (mRS = 3):130 (21%) Moderate to severe disability (mRS = 4): 41 (7%) Severe disability (mRS = 5–6): 21(3%)	avorable score (mRS = 0–2) :458 (72%) Moderate disability (mRS = 3):109(17%) Moderate to severe disability (mRS = 4): 44 (7%) Severe disability (mRS = 5–6): 21(3%)	oral; 20 mg once daily	6 months	Fluoxetine	Mortality, mRS, recurrent stroke, cardiac adverse event, thrombotic event, bleeding event
Hankey et al. (35)	AFFINITY	12-month follow-up report	2021	Australia, New Zealand, Vietnam	RCT	1, 280	64.05, 12.36	476, 804	6, 0.97			oral; 20 mg once daily	12 months	Fluoxetine	Mortality, mRS, recurrent stroke, cardiac adverse event
Bembenek et al. (22)	FOCUS-Poland	Primary report (6 and 12-month outcomes)	2020	Poland	RCT	61	66.67, 12.42	21, 40	5.58, 1.14	mRS = 0: 20 (66.7%) mRS = 1: 10 (33.3%) mRS = 2: 0(0)	mRS = 0: 19 (61.3%) mRS = 1: 10 (32.3%) mRS = 2: 2(6.5%)	oral; 20 mg once daily	12 months	Fluoxetine	Mortality, mRS, NIHSS
Kraglund et al. (20)	TALOS	Primary 6-month report	2018	Denmark	RCT	642	68, 13	221, 421	5.05, 5.21	mRS = 0: 262 (82%) mRS = 1: 31 (10%) mRS > = 2: 26 (8%)	mRS = 0: 261 (81%) mRS = 1: 36 (11%) mRS > = 2: 26(8%)	oral; 20 mg once daily(10 mg if aged ≥65 years or having reduced liver/kidney function)	6 months	Citalopram	Mortality, mRS, recurrent stroke, cardiac adverse event, bleeding event
Dennis et al.	FOCUS	Primary 6-month report	2019	UK	RCT	3, 127	71.35, 12.25	1,205, 1922	6.70, 5.94	Favorable score (mRS = 0–2) :572 (36%) Moderate disability (mRS = 3):518 (33%) Moderate to severe disability (mRS = 4): 121 (8%) Severe disability (mRS = 5–6): 342 (22%)	Favorable score (mRS = 0–2) :588 (38%) Moderate disability (mRS = 3):510 (33%) Moderate to severe disability (mRS = 4): 122(8%) Severe disability (mRS = 5–6): 333 (21%)	oral; 20 mg once daily	6 months	Fluoxetine	Mortality, recurrent stroke,mRS, cardiac adverse event, thrombotic event, bleeding event
Robinson et al. (16)	Robinson et al. trial	Primary 12-month report	2008	USA	RCT	117	62.59, 13.51	42, 75	6.80, 2.52	NA	NA	oral;10 mg/d for patients <65 years and 5 mg/d for patients ≥65 years	12 months	Escitalopram	cardiac adverse event, bleeding event
He et al. (19)	He et al. trial	36-month recurrence follow-up report	2018	China	RCT	404	61.93, 11.21	121, 283	5.68, 3.39	NA	NA	oral; 20 mg once daily	36 months	Fluoxetine	Mortality, recurrent stroke
He et al. (17)	He et al. trial	Primary 6-month functional-outcome report	2016	China	RCT	350	61.53, 11.06	101, 249	7.24, 3.47	NA	NA	oral; 20 mg once daily	6 months	Fluoxetine	Mortality, NIHSS

### 3.2 Risk of bias

The results of the risk-of-bias assessment are presented in Supplementary Figure S1. Two included RCTs, Yi-Tao He et al. (17) and Yitao He et al. (19), were classified as having a high risk of bias. The main concerns were related to allocation concealment, blinding of participants and personnel, and blinding of outcome assessment. These domains may introduce selection, performance, and detection bias, especially for functional and scale-based outcomes. The remaining studies (14, 16, 18, 20–25) were considered to have a low risk of bias. Because the two high-risk trials contributed data to some outcome-specific analyses, their potential influence was considered when interpreting the pooled estimates. In particular, Yi-Tao He et al. (17) contributed to the absNIHSS analysis, whereas Yitao He et al. (19) contributed to recurrent stroke outcomes. Therefore, findings involving these outcomes were interpreted cautiously, especially when substantial heterogeneity or a limited number of contributing studies was present. A formal quantitative sensitivity analysis excluding the two high-risk trials was not conducted because each trial contributed to a different outcome domain and its exclusion would leave only one study or a very small number of studies in some follow-up-duration subgroups. Such *post hoc* pooled estimates were considered insufficiently stable for meaningful interpretation. Instead, the potential influence of these trials was assessed qualitatively. Accordingly, the absNIHSS findings and recurrent stroke findings involving these trials were interpreted cautiously, particularly where statistical heterogeneity was substantial or the number of contributing studies was limited.

### 3.3 Primary outcomes: recurrent stroke, modified Rankin scale (mRS), absolute decrease of national institute of health stroke scale (absNIHSS)

#### 3.3.1 Overall outcomes of recurrent stroke

**Figure 2:**
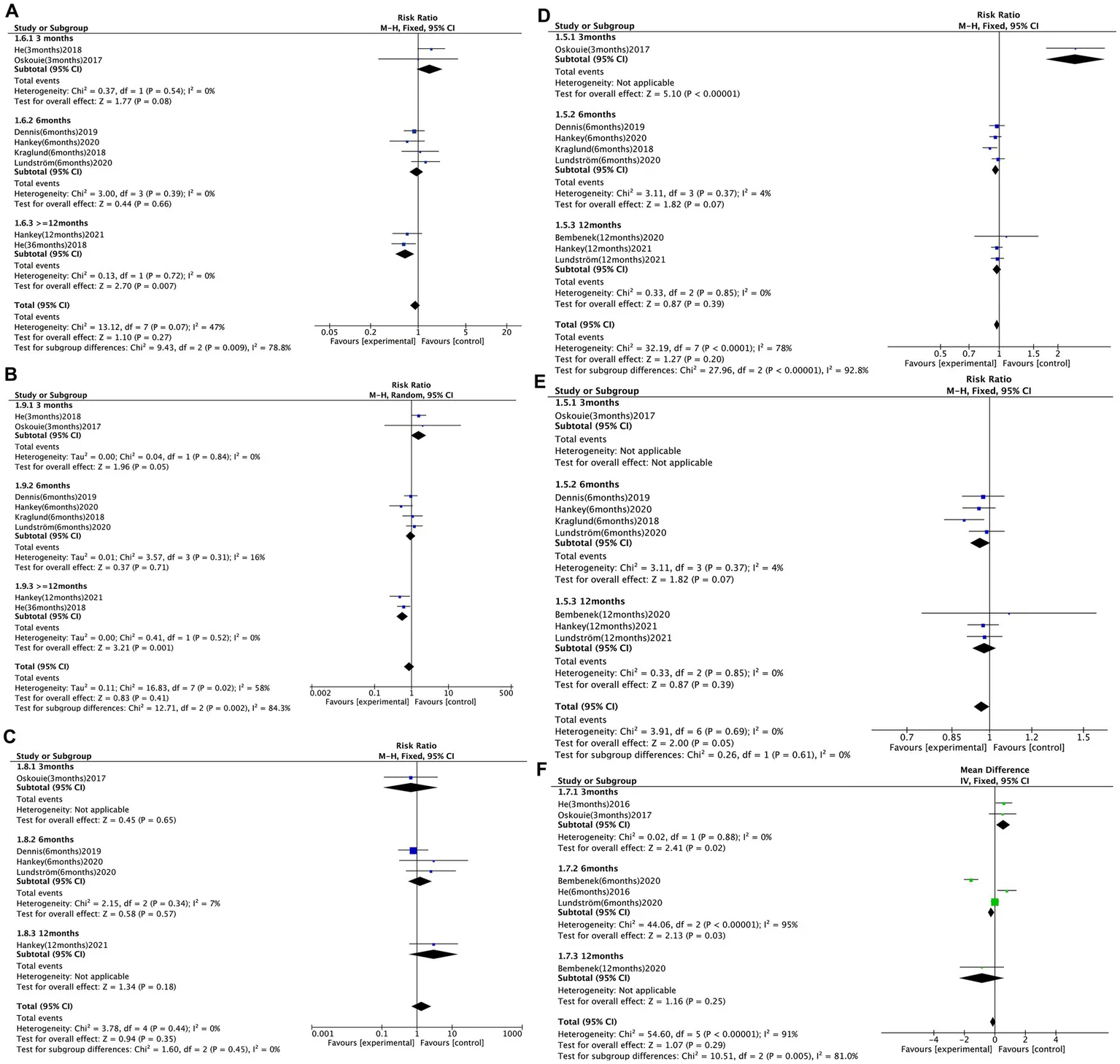
Forest plot of the meta-analyses evaluating the effect of SSRIs compared to control on **(A)** recurrent stroke **(B)** recurrent ischemic stroke **(C)** recurrent hemorrhagic stroke **(D)** modified Rankin scale (mRS) **(E)** mRS 6 and 12 months **(F)** absolute decrease of National Institute of Health stroke scale (absNIHSS). SSRIs, selective serotonin reuptake inhibitors; 95% CI, 95% confidence interval; RR, risk ratio.

Six RCTs (18–21, 23, 24), comprising 7,076 patients, explicitly reported the outcome of recurrent stroke. All of the RCTs (18–21, 23, 24) reported ischemic stroke and four of them (18, 21, 23, 24) reported hemorrhagic stroke. Consequently, they were included in the subgroup quantitative meta-analysis. The primary analysis encompassed all six RCTs, and while the pooled results indicated a trend toward a reduced risk of recurrent stroke in the SSRI group compared with the placebo group, this difference did not reach statistical significance using a fixed-effect model (RR 0.91, 95% CI 0.76–1.08; *p* = 0.27; *I*^2^ = 47%; Figure 2A). Subgroup analysis suggested that the effect estimates for recurrent stroke differed across follow-up duration categories (*p* = 0.009; Chi^2^ = 9.43; Figure 2A). However, these findings were exploratory and should not be interpreted as evidence that SSRIs causally reduce recurrent stroke in a time-dependent manner.

In the 3-month follow-up analysis, the SSRI group showed a non-significant higher risk compared with the control group using a fixed-effect model (2 RCTs, 274 patients, RR 1.48, 95% CI 0.96–2.29, *p* = 0.08, *I*^2^ = 0%, Figure 2A) (18, 19). However, when examining the 6-month study, the risk in the experimental group decreased and was found to be lower than that in the control group (RR 0.95, 95% CI 0.75–1.20; *p* = 0.66; *I^2^* = 0%; Figure 2A). In studies with follow-up equal to or longer than 12 months, the pooled estimate favored the SSRI group for recurrent stroke using a fixed-effect model (RR 0.65, 95% CI 0.47–0.89; *p* = 0.007; *I*^2^ = 0%; Figure 2A). This result indicates an association in the long-term follow-up subgroup, but it does not establish a causal temporal effect. This apparent long-term association may also reflect differences in enrolled populations, follow-up completeness, outcome ascertainment, secondary prevention strategies, and the greater statistical weight of large multicenter trials.

#### 3.3.2 Recurrent ischemic stroke of patients

Six RCTs (18–21, 23, 24), comprising 7,076 patients, explicitly reported the outcome of recurrent ischemic stroke. In terms of recurrent ischemic stroke, SSRIs showed a non-significant trend toward risk reduction using a random-effects model because heterogeneity was substantial (RR 0.87, 95% CI 0.64–1.20; *p* = 0.41; *I*^2^ = 58%; Figure 2B). Through subgroup analysis, we observed that the pooled estimates for recurrent ischemic stroke differed across follow-up duration categories (*p* = 0.002; Chi^2^ = 12.71; Figure 2B). Although the point estimates appeared lower in longer follow-up subgroups, this pattern should be interpreted cautiously because the subgroup analyses were exploratory and based on different study populations and follow-up designs.

At the 3-month follow-up, the SSRI group showed a borderline higher risk compared with the control group using a fixed-effect model (RR 1.57, 95% CI 1.00–2.48; *p* = 0.05; *I*^2^ = 0%; Figure 2B). Because this was a borderline finding, it should be interpreted cautiously. However, in the 6-month study, the risk in the experimental group decreased and was found to be lower than that in the control group (RR 0.95, 95% CI 0.70–1.27; *p* = 0.71; *I*^2^ = 16%; Figure 2B). In the analysis involving a follow-up period equal to or greater than 12 months, SSRI use was associated with a lower risk of recurrent ischemic stroke (RR 0.57, 95% CI 0.40–0.80; *p* = 0.001; *I*^2^ = 0%; Figure 2B). This finding should be regarded as a follow-up-duration-stratified association rather than proof of a time-dependent protective effect. The lower point estimate in the longer follow-up subgroup should therefore be interpreted as an observed association that requires confirmation, not as evidence that SSRIs exert a delayed biological protective effect.

#### 3.3.3 Recurrent hemorrhagic stroke of patients

In four RCTs (18, 21, 23, 24) comprising 6,030 patients, the outcome of recurrent hemorrhagic stroke was explicitly reported. Regarding recurrent hemorrhagic stroke, the observed increase in risk was not statistically significant using a fixed-effect model (RR 1.35, 95% CI 0.72–2.52; *p* = 0.35; *I*^2^ = 0%; Figure 2C). Through subgroup analysis, the point estimates for recurrent hemorrhagic stroke appeared higher in longer follow-up categories; however, the between-subgroup difference was not statistically significant (*p* = 0.45; Chi^2^ = 1.60; Figure 2C). Therefore, no firm conclusion can be drawn regarding a duration-related increase in hemorrhagic stroke risk.

At the 3-month follow-up, the experimental group exhibited a lower risk compared to the control group (RR 0.67, 95% CI 0.11–3.87; *p* = 0.65; Figure 2C). Meanwhile, in the 6-month study, the risk in the experimental group increased and was found to be higher than that in the control group (RR 1.25, 95% CI 0.59–2.66; *p* = 0.57; *I*^2^ = 7%; Figure 2C). In the analysis involving a follow-up period of 12 months, the risk in the experimental group continued to increase (RR 2.98, 95% CI 0.60–14.72; *p* = 0.18; Figure 2C).

#### 3.3.4 Modified rankin scale (mRS)

In stroke trials, we often use the modified Rankin Scale (mRS) to evaluate the prognosis of patients, with a score of 0 to 2 conventionally considered to represent independence, and individuals falling into this category are considered responders. Eight RCTs (14, 18, 20–25), comprising 6,733 patients, explicitly reported the outcome of mRS. The pooled results indicated that there was no statistically significant difference between the SSRI and control groups in terms of mRS outcomes using a random-effects model because heterogeneity was substantial (RR 0.98, 95% CI 0.95–1.01; *p* = 0.20; *I*^2^ = 78%; Figure 2D). The substantial heterogeneity indicates that the overall mRS estimate should be interpreted cautiously and that a single summary estimate may not fully represent the variability across trials. Potential explanations for this heterogeneity include differences in follow-up duration, baseline stroke severity, timing of SSRI initiation, rehabilitation background, mRS responder definitions, trial size, and SSRI regimens. In particular, large multicenter trials may have weighted the pooled estimate toward more neutral effects, whereas smaller trials may have contributed more variable estimates. Sensitivity analysis identified Oskouie et al. (18) as the primary contributor to heterogeneity. This study reported a higher proportion of patients achieving functional independence after SSRI use at 3 months (RR 2.46, 95% CI 1.74–3.48; *p* < 0.00001; Figure 2D). Importantly, this apparent short-term mRS benefit was derived entirely from this single trial, which was also the main source of heterogeneity. Therefore, the 3-month mRS finding should be regarded as a single-study signal rather than a stable pooled estimate or a generalizable clinical conclusion. In contrast, the 6-month and 12-month subgroups did not show statistically significant improvement in mRS outcomes (6 months, RR 0.96, 95% CI 0.92–1.00, *p* = 0.07, *I*^2^ = 4%, 12 months, RR 0.98, 95% CI 0.93–1.03; *p* = 0.39; *I*^2^ = 0%; Figure 2D). Therefore, the available evidence does not support a consistent mRS benefit across follow-up durations.

After excluding the study responsible for heterogeneity, heterogeneity was reduced to 0%, and the pooled estimate no longer suggested a clear mRS benefit using a fixed-effect model (RR 0.97, 95% CI 0.94–1.00; *p* = 0.05; *I*^2^ = 0%; Figure 2E). Because this result was borderline, it should not be interpreted as definitive evidence of either benefit or harm. This sensitivity analysis further indicates that the apparent short-term mRS improvement was mathematically dependent on a single heterogeneous study. Therefore, heterogeneity in the mRS analysis appears to reflect both statistical influence from an individual trial and clinical differences across studies rather than a consistent treatment effect across all included RCTs. Through subgroup analysis (*p* = 0.61; Chi^2^ = 0.26; Figure 2E), we observed that both the result of 6 months (RR 0.96, 95% CI 0.92–1.00; *p* = 0.07; *I^2^* = 4%; Figure 2E) and the 12 months (RR 0.98, 95% CI 0.93–1.03; *p* = 0.39; *I^2^* = 0%; Figure 2E) indicated an increase in mRS scores associated with SSRIs, though these increases were not statistically significant.

#### 3.3.5 Absolute decrease of national institute of health stroke scale (absNIHSS)

Incorporating data from four RCTs (17, 18, 22, 24) comprising 2034 patients, we explicitly examined the outcome related to absolute NIHSS reduction. The combined results suggested no statistically significant overall difference in absNIHSS between the SSRI and control groups using a random-effects model because heterogeneity was substantial (WMD −0.10, 95% CI − 0.30–0.09; *p* = 0.29; *I*^2^ = 91%; Figure 2F). Because heterogeneity was substantial, the pooled absNIHSS estimate should be interpreted cautiously and should not be used to infer a consistent functional effect across trials. The high heterogeneity may be related to variability in baseline neurological impairment, timing of NIHSS assessment, rehabilitation intensity, SSRI type and dose, treatment duration, and differences in how absolute NIHSS reduction was reported or calculated across trials. Furthermore, this analysis included one trial judged to be at high risk of bias, which further reduces the certainty of the absNIHSS finding. Therefore, the absNIHSS results should be considered exploratory rather than confirmatory. By conducting subgroup analysis, we found that the pooled estimates for absNIHSS differed across follow-up duration categories (*p* = 0.005; Chi^2^ = 10.51; Figure 2F). Given the limited number of studies and substantial heterogeneity, particularly in the 6-month subgroup, this finding should be interpreted as exploratory rather than as evidence of a causal time-dependent effect. The apparent change in direction across follow-up categories may be explained by differences in baseline neurological severity, NIHSS assessment timing, rehabilitation intensity, missing outcome data, attrition, SSRI regimen, and trial design.

At 3 months, SSRI use was associated with a greater absolute NIHSS reduction (WMD 0.57, 95% CI 0.11–1.03; *p* = 0.02; *I*^2^ = 0%; Figure 2F). At 6 months, SSRI use was associated with a smaller absolute NIHSS reduction using a random-effects model because heterogeneity was very high (WMD −0.23, 95% CI − 0.45 to −0.02; *p* = 0.03; *I*^2^ = 95%; Figure 2F). However, given the limited number of studies and substantial heterogeneity in the 6-month subgroup, these findings should be interpreted as exploratory and inconsistent rather than as evidence of a reliable functional trajectory. The very high heterogeneity in this subgroup suggests that the 6-month absNIHSS estimate may be sensitive to between-trial differences and should not be interpreted as a uniform mid-term effect of SSRI therapy. Accordingly, the mid-term absNIHSS finding should not be interpreted as evidence of harm or a true loss of biological efficacy over time. In the 12-month study, the reduction in the experimental group continued to decrease, although this change did not reach statistical significance (WMD −0.85, 95% CI −2.29–0.59; *p* = 0.25; Figure 2F).

### 3.4 Secondary outcomes: bleeding event, cardiac adverse event, death event, thrombotic event, effect of various drugs

Our meta-analysis showed no statistically significant difference in bleeding events using a fixed-effect model (RR 1.19, 95% CI 0.87–1.63; *p* = 0.27; *I*^2^ = 0%), cardiac adverse events using a fixed-effect model (RR 0.92, 95% CI 0.71–1.17; *p* = 0.49; *I*^2^ = 0%), or death events using a fixed-effect model (RR 1.04, 95% CI 0.88–1.23; *p* = 0.65; *I*^2^ = 0%) between the SSRI and placebo groups. Both in the overall analysis and in subgroup analyses at 3 months, 6 months, and 12 months. Compared with the control group, the SSRI group showed a borderline non-significant trend toward fewer thrombotic events using a fixed-effect model (RR 0.77, 95% CI 0.60–1.00; *p* = 0.05; *I*^2^ = 0%). Given the borderline *p* value and confidence interval reaching 1.00, this result should be interpreted cautiously. Our sub-analysis of the effects of fluoxetine and other drugs also showed no statistically significant difference among different drugs (bleeding event: Chi^2^ = 0.14, *p* = 0.71; death event: Chi^2^ = 0.53, *p* = 0.47; mRS: Chi^2^ = 3.85, *p* = 0.05). Further studies in the future will be necessary to confirm these findings. Detailed analyses for these outcomes can be found in Supplementary Figure S2 and Supplementary Figure S3.

## 4 Discussion

Our analysis suggested several exploratory findings. The short-term mRS signal was derived entirely from a single trial and should therefore be interpreted as a single-study signal rather than evidence of a robust functional benefit. The absNIHSS findings were inconsistent across follow-up categories, with a favorable signal at 3 months but an unfavorable finding at 6 months. Long-term follow-up subgroups showed an association with lower recurrent stroke risk, particularly recurrent ischemic stroke. However, no statistically significant differences were observed for bleeding events, mortality, cardiac adverse events, thrombotic events, or drug-specific subgroup analyses. Overall, these findings should be interpreted as follow-up-duration-stratified associations rather than evidence of a causal biological time-dependent effect of SSRIs.

Although SSRIs have established efficacy for post-stroke depressive symptoms, their prognostic effects on neurological recovery remain uncertain. Possible biological mechanisms, including neuroplasticity, neurogenesis, microvascular support, retinoic acid metabolism, and endoplasmic reticulum stress-related pathways, may partly explain why SSRIs have been investigated for stroke recovery. However, these mechanisms remain speculative in relation to the present clinical findings. The current meta-analysis cannot determine whether these biological pathways explain the observed subgroup patterns. Therefore, mechanistic explanations should be separated from evidence-based conclusions and regarded as hypothesis-generating rather than confirmatory.

Despite the high prevalence of depression in patients with stroke and the proven efficacy of SSRIs in treating depressive symptoms in that population, the effects on prognosis outcomes of stroke and their mechanisms have remained unclear. SSRIs have demonstrated the ability to influence critical cellular processes related to neuronal cell survival and neuroplasticity, implying their potential utility in the treatment of neurological disorders like stroke, which may exert an influence on prognosis results (26). Several mechanisms have been proposed to explain the potential benefits of SSRIs in stroke recovery in current studies: promoting enhancement of neurogenesis, neural cell migration, and microvessel support in the peri-infarct region of the ischemic brain (27); inhibiting retinoic acid (RA) catabolism (28); targeting human proteins such as neurogenic locus notch homolog protein 1 (NOTCH 1) and Rho-associated protein kinase 1 (ROCK 1), which may act as promising regulators in neurorecovery (29); restoring inhibitory neural network tonus to facilitate motor rehabilitation (39); attenuating endoplasmic reticulum stress (ERS)-induced neuron apoptosis by reducing the expression of GRP-78, caspase-12, CHOP, and caspase-3 (30) etc. However, the specific contributions of these mechanisms to stroke prognosis require further exploration and investigation.

Our findings are broadly consistent with previous meta-analyses (10), showing no robust overall improvement in functional independence defined as mRS 0–2 after SSRI use. Although a short-term mRS signal was observed, it was based on a single trial and should not be interpreted as a generalizable functional benefit. The absNIHSS findings require particular caution. Although the 3-month subgroup suggested greater NIHSS reduction, the 6-month subgroup showed a smaller NIHSS reduction in the SSRI group and very high heterogeneity. This mid-term finding does not support a sustained neurological benefit and should not be interpreted as definitive evidence of harm. Instead, it may reflect differences in baseline stroke severity, NIHSS assessment timing, rehabilitation exposure, missing data, treatment duration, and trial design.

A possible explanation for the observed heterogeneity is that the included trials differed substantially in clinical and methodological characteristics. The SSRI regimens were not identical, with most patients receiving fluoxetine and fewer receiving citalopram or escitalopram. Treatment timing, dose, and duration also varied across trials. In addition, baseline stroke severity, rehabilitation exposure, outcome definitions, and follow-up schedules were not fully standardized. The large multicenter trials, including EFFECTS, AFFINITY, and FOCUS, accounted for a substantial proportion of the total sample size and may have driven pooled estimates toward more neutral effects, whereas smaller trials contributed more variable estimates. Thus, heterogeneity should be interpreted as reflecting both clinical diversity and methodological variation, rather than a uniform biological effect of SSRIs.

Although the follow-up-duration-stratified analyses suggested different patterns across short-, mid-, and long-term outcomes, these findings should not be interpreted as confirming a biological time-dependent effect of SSRIs. Alternative explanations are plausible, including differences in study populations, baseline stroke severity, follow-up schedules, outcome definitions, rehabilitation strategies, missing outcome data, attrition, and the disproportionate contribution of large multicenter RCTs. Therefore, the observed subgroup patterns should be regarded as exploratory associations rather than evidence of a causal temporal mechanism.

While recent research has identified an association between SSRIs and neuroplasticity, the clinical time course of any potential effect remains uncertain. Previous experimental and clinical studies have suggested that SSRIs may influence neuroplasticity and motor recovery, but the present meta-analysis cannot determine whether these mechanisms explain the different findings observed across follow-up subgroups. Therefore, mechanistic explanations for short-, mid-, or long-term differences should be considered hypothesis-generating rather than confirmatory. In patients with ischemic stroke, earlier administration of SSRIs can also improve neurological functional prognosis and decrease copeptin levels, whereas long-term use may be associated with some adverse effects such as seizures and fractures (11, 12). These factors could underlie the observed effects on functional independence. Until now, there have been studies delving into this matter, yielding conflicting results. A prospective observational study involving patients with acute ischemic stroke demonstrated that early sertraline treatment tended to enhance functional recovery within 3 months (12). Conversely, another observational study indicated that SSRI therapy did not lead to improvements in disability and quality of life for multi-ethnic Asian patients experiencing their first-ever stroke (13). Moreover, ongoing studies are actively investigating both the acute and long-term effects, as well as determining the appropriate dosage for administering SSRIs in stroke patients (NCT01937182, NCT04221256, and NCT02767999), and we eagerly anticipate their outcomes. Further studies and RCTs on this topic are necessary to better clarify and support our results.

On the other hand, although the overall result for recurrent stroke did not reach statistical significance, the long-term follow-up subgroup showed an association between SSRI use and a lower risk of recurrent stroke, particularly recurrent ischemic stroke. This finding may suggest a potential long-term association, but it should not be interpreted as definitive evidence of a time-dependent protective effect because the subgroup analyses were exploratory and involved different study populations and follow-up designs. In addition, long-term estimates may be influenced by attrition, missing follow-up data, differential outcome ascertainment, secondary prevention strategies, and the disproportionate contribution of large multicenter RCTs. Chollet et al. proposed that fluoxetine may influence the serotonergic system and long-term potentiation, providing a possible mechanistic rationale for further research. However, the present meta-analysis does not establish that this mechanism explains the long-term recurrent stroke association. The apparent discrepancy between mid-term functional outcomes and long-term recurrent stroke findings should be interpreted cautiously. Rather than indicating a true temporal biological pattern, it may reflect differences in outcome type, study populations, statistical power, follow-up design, missing data, and trial weighting. Further randomized controlled trials are needed to determine whether any duration-related association is reproducible. Another finding was that the point estimates differed between recurrent ischemic and hemorrhagic stroke outcomes across follow-up subgroups. However, these patterns were not consistent across all outcomes, and the hemorrhagic stroke results were not statistically significant. Therefore, these observations should be interpreted cautiously and should not be described as a difference across follow-up duration categories. Irene Lopez-Vilchez et al. (31) discovered that SSRIs could inhibit thrombin-induced platelet response by interfering with cytoskeletal assembly and associated signaling pathways, offering a possible explanation for the recurrence outcomes seen in two distinct stroke types. Nevertheless, the reasons why the observed associations differed across follow-up-duration categories remain unclear. These differences may reflect variations in study populations, baseline stroke severity, treatment timing, duration of SSRI exposure, outcome definitions, follow-up schedules, rehabilitation strategies, missing data, attrition over time, and statistical power, rather than a true biological time-dependent effect. Therefore, the subgroup findings should be interpreted as exploratory associations and not as evidence for an established temporal mechanism. Furthermore, the dominance of large multicenter fluoxetine trials, such as EFFECTS, AFFINITY, and FOCUS, may have influenced pooled estimates toward neutral results, whereas smaller trials with different designs, SSRI regimens, and patient characteristics may have contributed more variable effect estimates. This imbalance should be considered when interpreting both overall and subgroup findings.

Our analysis of bleeding events, death events, cardiac adverse events, thrombotic events, and drug-specific subgroups showed no statistically significant differences between SSRI and control groups, which is consistent with previous reviews (10). However, when compared to the control group, the experimental group had a lower rate of thrombotic events, though this was not statistically significant. Ofek et al. (32) proved that fluoxetine induces vasodilatation of cerebral arterioles by co-modulating NO/muscarinic signaling to improve motor, cognitive and executive functions. Additionally, a prior review on the cerebrovascular effects of SSRIs, which incorporated WHO data and several studies, concluded that SSRI treatment was associated with a very low rate of cerebrovascular adverse reactions (33). To provide more conclusive evidence, appropriately powered RCTs are required to investigate the impact of SSRIs on these outcomes.

The updated search from 1 July 2022 to 7 July 2026 did not identify additional randomized controlled trials eligible for inclusion in our quantitative synthesis. However, newer evidence has continued to address SSRI-related vascular and safety outcomes. A recent population-based retrospective cohort study and meta-analysis compared the risk of stroke among individuals receiving SSRIs versus non-SSRI antidepressants (34). Although this study was not eligible for inclusion in the present RCT-based meta-analysis, it provides relevant observational context and highlights the importance of considering antidepressant selection, baseline vascular risk, and confounding by indication when interpreting recurrent stroke outcomes. In addition, a recent systematic review and meta-analysis examined fracture risk in stroke patients treated with SSRIs (35) This evidence is clinically relevant because fracture risk may influence the overall risk–benefit profile of SSRI therapy after stroke, particularly in older patients, patients with baseline disability, and those at high risk of falls. Therefore, although the updated search did not change our pooled estimates, emerging evidence reinforces the need for individualized clinical decision-making and further randomized trials assessing both efficacy and long-term safety.

Our findings have potential clinical implications, but they should be applied cautiously. The current evidence does not support routine short-term or long-term SSRI therapy solely for improving post-stroke prognosis. The apparent short-term functional signal was driven by a single trial, and the mid-term absNIHSS findings did not support sustained neurological benefit. Although long-term follow-up subgroups suggested a lower risk of recurrent stroke, these findings were exploratory and may reflect differences in trial design, patient characteristics, secondary prevention, and follow-up completeness. Therefore, the optimal duration of SSRI therapy after stroke remains uncertain and should be determined according to the approved indication, patient-level risk–benefit profile, psychiatric comorbidity, potential adverse effects, and drug interactions. Future RCTs should directly compare different treatment durations and define clinically meaningful functional and safety endpoints.

### 4.1 Advantages and limitations

Our study provides a comprehensive review of the various aspects of SSRI prognostic results in post-stroke patients. We have elucidated the impact of SSRIs on post-stroke patients with regard to patient-scale scores and clinical characteristics, and we have undertaken a subgroup analysis based on time. Assessing both short-term effectiveness and the potential follow-up duration-stratified findings holds promise for guiding clinical practice and laying the groundwork for future research.

Given the potential clinical implications of the results of our meta-analysis, it is crucial to transparently acknowledge its limitations.

One important limitation is that 12 potentially relevant records could not be retrieved in full text despite repeated retrieval attempts. Exclusion of unavailable full-text records may introduce selection bias, particularly if these studies differed systematically from included trials in geographic region, publication type, language, sample size, study quality, or direction of results. Because eligibility, intervention details, outcome definitions, risk of bias, and numerical data could not be verified, quantitative sensitivity analysis was not feasible. Therefore, their potential influence was assessed qualitatively using available title, abstract, bibliographic, and indexing information. This limitation should be considered when interpreting the completeness, robustness, and generalizability of the pooled estimates.

Firstly, it should be noted that the majority of patients in our review received fluoxetine, escitalopram, or citalopram, with fluoxetine accounting for 89.8% of cases. The generalizability of our findings to other SSRIs remains uncertain. Although we found no statistically significant difference between the effects of fluoxetine and other SSRIs, the subjects were too small to yield reliable results. Currently, fluoxetine has been shown to facilitate brain repair and enhance clinical outcomes through its anti-inflammatory effect even years after stroke onset, suggesting potential superiority over other SSRIs (8, 36). However, our study did not reveal a significant statistical effect due to its constrained scope. Moreover, our analyses suggested that some pooled estimates differed across follow-up-duration categories. However, these findings were exploratory and cannot determine whether the effects of SSRIs are dose-dependent, time-dependent, or carryover-effect-dependent. Because aggregate study-level data were used, we could not adequately assess individual-level changes over time, missing data mechanisms, attrition, rehabilitation exposure, baseline stroke severity, or interactions between treatment duration and patient characteristics. Further studies with individual patient data, standardized follow-up schedules, and pre-specified subgroup analyses are required to clarify these questions.

Second, the absence of raw individual patient data made exploratory subgroup analyses stratified by stroke severity, physical examination indicators after recurrence, and dose of implication impossible. Consequently, it may be necessary to delve into the specifics of our results to uncover the underlying interconnected mechanisms, which should be subject to further investigation to explore and validate our findings.

Thirdly, all our SSRI trials included in this meta-analysis have strict inclusion criteria. Since all our adopted studies included only stroke patients without other complications or other merged diseases, our results should be considered valid only for this specific population. Therefore, patients with common complications such as infection, edema, embolism, and significant comorbid symptoms such as post-stroke fatigue (PSF), central poststroke pain (CPSP), and post-stroke depression (PSD) would be excluded from these trials. It’s worth acknowledging that numerous studies have highlighted the benefits of SSRIs for patients with additional comorbidities. However, it is plausible that these medications may have varying effects on prognosis in the presence of different diseases (37, 38). Moreover, the majority of the studies or patients in our analysis were of Caucasian ethnicity, and the available patient information was not entirely comprehensive. This aspect may introduce potential biases when extrapolating our study’s outcomes to other racial groups, warranting further exploration.

There was also substantial clinical and statistical heterogeneity in the mRS analysis. The apparent 3-month mRS benefit was driven entirely by Oskouie 2017 (18), which was the only trial reporting short-term mRS outcomes and the primary source of heterogeneity. After excluding this trial, heterogeneity was markedly reduced and the pooled mRS estimate no longer indicated a clear functional benefit. Therefore, conclusions regarding short-term mRS improvement are highly dependent on this single study and should be interpreted with caution. Despite our efforts to explore heterogeneity by categorizing follow-up duration into short-, mid-, and long-term periods, the observed differences across follow-up categories should be interpreted cautiously. Follow-up duration alone may not fully explain the observed heterogeneity. Other potential sources include SSRI type and dosage, timing of treatment initiation, intervention duration, baseline stroke severity, rehabilitation exposure, trial design, outcome definitions, risk of bias, and the disproportionate influence of large multicenter RCTs. Because aggregate study-level data were used, we could not perform individual patient data analyses or meta-regression to formally evaluate these sources of heterogeneity. In particular, subgroup analyses with only one contributing study cannot provide robust pooled evidence and may reflect trial-specific characteristics rather than a reproducible treatment effect. These subgroup findings may reflect differences in trial design, sample size, patient characteristics, treatment timing, intervention duration, outcome definitions, or follow-up schedules rather than true temporal effects of SSRIs. They may reflect missing outcome data, attrition over longer follow-up, heterogeneity in rehabilitation strategies, differences in SSRI selection and dosing strategies, baseline neurological severity, concurrent rehabilitation, and the relative weight of large multicenter trials in pooled analyses. Therefore, the follow-up-duration subgroup findings should be considered exploratory and hypothesis-generating. They may also reflect differences in SSRI selection, dosing strategies, baseline neurological severity, concurrent rehabilitation, and the relative weight of large multicenter trials in pooled analyses. Therefore, subgroup findings should be considered exploratory and hypothesis-generating. We did not re-assess the included trials using the newer Cochrane RoB 2 tool, which provides a structured domain-based assessment of bias arising from the randomization process, deviations from intended interventions, missing outcome data, outcome measurement, and selective reporting. RoB 1 was retained because it had been adopted in the original protocol and review process and was applied consistently across all included trials. This approach avoided *post hoc* changes in risk-of-bias judgment criteria during manuscript revision. Nevertheless, the absence of a full RoB 2 reassessment is a methodological limitation. To address this limitation, we reported the RoB 1 domain-level concerns, explicitly identified high-risk trials, and interpreted outcome estimates involving these trials with caution. We did not perform a separate quantitative sensitivity analysis excluding trials judged to be at high risk of bias. Because only one high-risk trial contributed to each affected outcome domain, and because exclusion would leave only one study or a very small number of studies in some follow-up-duration subgroups, the resulting post hoc pooled estimates were considered statistically unstable and potentially misleading. The influence of these trials was therefore assessed qualitatively, and the certainty of the corresponding findings should be interpreted cautiously. Future updates of this review should prospectively apply RoB 2 and prespecified sensitivity analyses excluding high-risk trials. Additionally, due to the limited number of studies included in several outcome-specific analyses, there may not have been enough studies to adequately assess publication bias. Although we updated the literature search from 1 July 2022 to 7 July 2026, no additional eligible randomized controlled trials were identified. Therefore, the main findings and conclusions remained unchanged. Nevertheless, emerging evidence may continue to influence the interpretation of SSRI effects on post-stroke prognosis, particularly regarding patient selection, treatment timing, intervention duration, functional outcomes, and adverse events.

## 5 Conclusion

Exploratory analyses stratified by follow-up duration did not demonstrate a consistent functional benefit of SSRIs after stroke. The apparent 3-month mRS benefit was derived from a single trial, whereas the absNIHSS findings were inconsistent and showed substantial heterogeneity. Although the ≥12-month subgroup was associated with lower risks of recurrent stroke and recurrent ischemic stroke, these estimates were derived from exploratory comparisons across trials and do not establish a causal temporal or biological effect. No statistically significant differences were observed in bleeding events, mortality, cardiac adverse events, thrombotic events, or drug-specific subgroup analyses. Further adequately powered randomized controlled trials and individual participant data meta-analyses are needed to clarify the prognostic role, optimal treatment duration, patient selection, and safety of SSRIs after stroke.

## Data Availability

The original contributions presented in the study are included in the article/Supplementary material, further inquiries can be directed to the corresponding author.
