# Supplementary material for: The influence of selective serotonin reuptake inhibitors on stroke outcome: a systematic review and meta-analysis
Source: Front Neurol. 2026 Aug 26;17:1894976. doi: 10.3389/fneur.2026.1894976 (PMC13553457; doi:10.3389/fneur.2026.1894976)
Supplement: Supplementary file 1 [file Data_Sheet_1.DOCX]

## Supplementary Material

**Supplementary Fig. S1.** Detailed ROB assessment results.

**Supplementary Fig. S2. Forest plot of individual studies and meta-analysis. Results of effect of various drugs. (A) Bleeding event (B) Death event (C) mRS (D) Recurrent stroke.**

Abbreviation: SSRIs, Selective serotonin reuptake inhibitors; 95% CI, 95% Confidence interval; RR, Risk ratio

**Supplementary Fig. S3 Forest plot of individual studies and meta-analysis. Results of effect between the SSRI and placebo groups.**

(A) Bleeding event (B) Death event (C) thrombotic event (D) cardiac adverse event.

Abbreviation: SSRIs, Selective serotonin reuptake inhibitors; 95% CI, 95% Confidence interval; RR, Risk ratio

**Supplementary Table S1.** Search strategy and resulting net search formula.

We conducted an online electronic search of Cochrane library, PubMed, and Embase in June 2026 using free words and MeSH terms.

**(A) Search strategy**

|  | Pubmed | Embase | Cochrane | Combined |
| --- | --- | --- | --- | --- |
| MeSH terms | Serotonin Uptake Inhibitors | serotonin uptake inhibitor | Serotonin Uptake Inhibitors | 5 HT Uptake Inhibitor* |
| Free words | Reuptake Inhibitors, Serotonin | antidepressants, serotonin specific reuptake inhibitors | 5 Hydroxytryptamine Uptake Inhibitor | 5 Hydroxytryptamine Uptake Inhibitor* |
|  | Uptake Inhibitors, 5-Hydroxytryptamine | selective serotonin reuptake inhibitor | Inhibitors, Serotonin Reuptake | antidepressants, serotonin specific reuptake inhibitors |
|  | Uptake Inhibitors, 5 Hydroxytryptamine | serotonin reuptake inhibitor | Inhibitor, 5-HT Uptake | Selective Serotonin Reuptake Inhibitor* |
|  | Uptake Inhibitors, Serotonin | serotonin specific reuptake inhibitor | Inhibitor, 5-Hydroxytryptamine Uptake | Serotonin Reuptake Inhibitor* |
|  | Serotonin Reuptake Inhibitor | serotonin specific reuptake inhibitors | 5 Hydroxytryptamine Uptake Inhibitors | serotonin specific reuptake inhibitor* |
|  | Inhibitor, Serotonin Reuptake | serotonin uptake inhibitors | Inhibitors, 5-Hydroxytryptamine Uptake | Serotonin Uptake Inhibitor* |
|  | Reuptake Inhibitor, Serotonin | SSRI | 5-Hydroxytryptamine Uptake Inhibitor | SSRI |
|  | Serotonin Uptake Inhibitor | SSRI antidepressant | Uptake Inhibitor, Serotonin | SSRI antidepressant |
|  | Inhibitor, Serotonin Uptake |  | Uptake Inhibitors, 5 Hydroxytryptamine |  |
|  | Uptake Inhibitor, Serotonin |  | 5-Hydroxytryptamine Uptake Inhibitors |  |
|  | 5-HT Uptake Inhibitor |  | Inhibitors, 5-HT Uptake |  |
|  | 5 HT Uptake Inhibitor |  | Uptake Inhibitors, 5-Hydroxytryptamine |  |
|  | Inhibitor, 5-HT Uptake |  | Reuptake Inhibitors, Serotonin |  |
|  | Uptake Inhibitor, 5-HT |  | Uptake Inhibitors, 5 HT |  |
|  | 5-Hydroxytryptamine Uptake Inhibitor |  | Reuptake Inhibitor, Serotonin |  |
|  | 5 Hydroxytryptamine Uptake Inhibitor |  | 5 HT Uptake Inhibitor |  |
|  | Inhibitor, 5-Hydroxytryptamine Uptake |  | Inhibitors, 5 Hydroxytryptamine Uptake |  |
|  | Uptake Inhibitor, 5-Hydroxytryptamine |  | Uptake Inhibitor, 5-Hydroxytryptamine |  |
|  | 5-HT Uptake Inhibitors |  | Inhibitors, 5 HT Uptake |  |
|  | 5 HT Uptake Inhibitors |  | Uptake Inhibitor, 5-HT |  |
|  | 5-Hydroxytryptamine Uptake Inhibitors |  | 5 HT Uptake Inhibitors |  |
|  | 5 Hydroxytryptamine Uptake Inhibitors |  | Serotonin Reuptake Inhibitors |  |
|  | Inhibitors, 5-HT Uptake |  | Inhibitor, Serotonin Reuptake |  |
|  | Inhibitors, 5 HT Uptake |  | 5-HT Uptake Inhibitors |  |
|  | Inhibitors, 5-Hydroxytryptamine Uptake |  | Uptake Inhibitors, Serotonin |  |
|  | Inhibitors, 5 Hydroxytryptamine Uptake |  | Serotonin Reuptake Inhibitor |  |
|  | Inhibitors, Serotonin Reuptake |  | Serotonin Uptake Inhibitor |  |
|  | Inhibitors, Serotonin Uptake |  | Inhibitors, Serotonin Uptake |  |
|  | Serotonin Reuptake Inhibitors |  | Inhibitor, Serotonin Uptake |  |
|  | Uptake Inhibitors, 5-HT |  | Uptake Inhibitors, 5-HT |  |
|  | Uptake Inhibitors, 5 HT |  | 5-HT Uptake Inhibitor |  |
|  | Selective Serotonin Reuptake Inhibitors |  | Selective Serotonin Reuptake Inhibitors |  |
|  | Selective Serotonin Reuptake Inhibitor |  | Selective Serotonin Reuptake Inhibitor |  |

**(A) Search strategy (continue)**

|  | Pubmed | Embase | Cochrane | Combined |
| --- | --- | --- | --- | --- |
| MeSH terms | Stroke | Stroke | Stroke | Acute Cerebrovascular Accident* |
| Free words | Acute Cerebrovascular Accident |  | Acute Cerebrovascular Accident | Acute Stroke* |
|  | Acute Cerebrovascular Accidents |  | Acute Cerebrovascular Accidents | Apoplexy |
|  | Acute Stroke |  | Acute Stroke | Brain Vascular Accident* |
|  | Acute Strokes |  | Acute Strokes | Cerebral Stroke* |
|  | Apoplexy |  | Apoplexy | Cerebrovascular Accident* |
|  | Apoplexy, Cerebrovascular |  | Apoplexy, Cerebrovascular | Cerebrovascular Apoplexy |
|  | Brain Vascular Accident |  | Brain Vascular Accident | Cerebrovascular Stroke* |
|  | Brain Vascular Accidents |  | Brain Vascular Accidents | CVA* Cerebrovascular Accident |
|  | Cerebral Stroke |  | Cerebral Stroke | Stroke* |
|  | Cerebral Strokes |  | Cerebral Strokes |  |
|  | Cerebrovascular Accident |  | Cerebrovascular Accident |  |
|  | Cerebrovascular Accident, Acute |  | Cerebrovascular Accident, Acute |  |
|  | Cerebrovascular Accidents |  | Cerebrovascular Accidents |  |
|  | Cerebrovascular Accidents, Acute |  | Cerebrovascular Accidents, Acute |  |
|  | Cerebrovascular Apoplexy |  | Cerebrovascular Apoplexy |  |
|  | Cerebrovascular Stroke |  | Cerebrovascular Stroke |  |
|  | Cerebrovascular Strokes |  | Cerebrovascular Strokes |  |
|  | CVA (Cerebrovascular Accident) |  | CVA Cerebrovascular Accident |  |
|  | CVAs (Cerebrovascular Accident) |  | CVAs Cerebrovascular Accident |  |
|  | Stroke, Acute |  | Stroke, Acute |  |
|  | Stroke, Cerebral |  | Stroke, Cerebral |  |
|  | Stroke, Cerebrovascular |  | Stroke, Cerebrovascular |  |
|  | Strokes |  | Strokes |  |
|  | Strokes, Acute |  | Strokes, Cerebral |  |
|  | Strokes, Cerebral |  | Strokes, Cerebrovascular |  |
|  | Strokes, Cerebrovascular |  | Synonyms: Strokes, Acute |  |
|  | Vascular Accident, Brain |  | Vascular Accident, Brain |  |
|  | Vascular Accidents, Brain |  | Vascular Accidents, Brain |  |

**(B) Resulting net search formula.**

***PubMed***

| Search number | Query | Search Details | Results | Time |
| --- | --- | --- | --- | --- |
| 5 | (#1 OR #2) AND (#3 OR #4) | ("acute cerebrovascular accident*"[Title/Abstract] OR "acute stroke*"[Title/Abstract] OR "Apoplexy"[Title/Abstract] OR "brain vascular accident*"[Title/Abstract] OR "cerebral stroke*"[Title/Abstract] OR "cerebrovascular accident*"[Title/Abstract] OR "Cerebrovascular Apoplexy"[Title/Abstract] OR "cerebrovascular stroke*"[Title/Abstract] OR "stroke*"[Title/Abstract] OR "stroke"[MeSH Terms]) AND ("5 ht uptake inhibitor*"[Title/Abstract] OR "5 hydroxytryptamine uptake inhibitor*"[Title/Abstract] OR "selective serotonin reuptake inhibitor*"[Title/Abstract] OR "serotonin reuptake inhibitor*"[Title/Abstract] OR "serotonin specific reuptake inhibitor*"[Title/Abstract] OR "serotonin uptake inhibitor*"[Title/Abstract] OR "SSRI"[Title/Abstract] OR "SSRI antidepressant"[Title/Abstract] OR "serotonin uptake inhibitors"[MeSH Terms]) | 630 | 03:19:38 |
| 4 | Serotonin Uptake Inhibitors[MeSH Terms] | "serotonin uptake inhibitors"[MeSH Terms] | 22,373 | 03:19:27 |
| 3 | "5 HT Uptake Inhibitor*"[Title/Abstract] OR "5 Hydroxytryptamine Uptake Inhibitor*"[Title/Abstract] OR "antidepressants, serotonin specific reuptake inhibitors"[Title/Abstract] OR "Selective Serotonin Reuptake Inhibitor*"[Title/Abstract] OR "Serotonin Reuptake Inhibitor*"[Title/Abstract] OR "serotonin specific reuptake inhibitor*"[Title/Abstract] OR "Serotonin Uptake Inhibitor*"[Title/Abstract] OR "SSRI"[Title/Abstract] OR "SSRI antidepressant"[Title/Abstract] | "5 ht uptake inhibitor*"[Title/Abstract] OR "5 hydroxytryptamine uptake inhibitor*"[Title/Abstract] OR "selective serotonin reuptake inhibitor*"[Title/Abstract] OR "serotonin reuptake inhibitor*"[Title/Abstract] OR "serotonin specific reuptake inhibitor*"[Title/Abstract] OR "serotonin uptake inhibitor*"[Title/Abstract] OR "SSRI"[Title/Abstract] OR "SSRI antidepressant"[Title/Abstract] | 21,376 | 03:19:08 |
| 2 | Stroke[MeSH Terms] | "stroke"[MeSH Terms] | 201,450 | 03:18:51 |
| 1 | "Acute Cerebrovascular Accident*"[Title/Abstract] OR "Acute Stroke*"[Title/Abstract] OR "Apoplexy"[Title/Abstract] OR "Brain Vascular Accident*"[Title/Abstract] OR "Cerebral Stroke*"[Title/Abstract] OR "Cerebrovascular Accident*"[Title/Abstract] OR "Cerebrovascular Apoplexy"[Title/Abstract] OR "Cerebrovascular Stroke*"[Title/Abstract] OR "CVA* Cerebrovascular Accident "[Title/Abstract] OR "Stroke*"[Title/Abstract] | "acute cerebrovascular accident*"[Title/Abstract] OR "acute stroke*"[Title/Abstract] OR "Apoplexy"[Title/Abstract] OR "brain vascular accident*"[Title/Abstract] OR "cerebral stroke*"[Title/Abstract] OR "cerebrovascular accident*"[Title/Abstract] OR "Cerebrovascular Apoplexy"[Title/Abstract] OR "cerebrovascular stroke*"[Title/Abstract] OR "stroke*"[Title/Abstract] | 403,245 | 03:18:27 |

***Cochrane***

| No. | Query | Results |
| --- | --- | --- |
| #1 | (‘Acute Cerebrovascular Accident*’ OR ‘Acute Stroke*’ OR ‘Apoplexy’ OR ‘Brain Vascular Accident*’ OR ‘Cerebral Stroke*’ OR ‘Cerebrovascular Accident*’ OR ‘Cerebrovascular Apoplexy’ OR ‘Cerebrovascular Stroke*’ OR ‘CVA* Cerebrovascular Accident ‘ OR ‘Stroke*’):ti,ab,kw | 87339 |
| #2 | MeSH descriptor: [Stroke] explode all trees | 19822 |
| #3 | (‘5 HT Uptake Inhibitor*’ OR ‘5 Hydroxytryptamine Uptake Inhibitor*’ OR ‘antidepressants, serotonin specific reuptake inhibitors’ OR ‘Selective Serotonin Reuptake Inhibitor*’ OR ‘Serotonin Reuptake Inhibitor*’ OR ‘serotonin specific reuptake inhibitor*’ OR ‘Serotonin Uptake Inhibitor*’ OR ‘SSRI’ OR ‘SSRI antidepressant’ ):ti,ab,kw | 7538 |
| #4 | MeSH descriptor: [Serotonin Uptake Inhibitors] explode all trees | 3557 |
| #5 | (#1 OR #2) AND (#3 OR #4) | 186 |

***Embase***

| No. | Query | Results | Date |
| --- | --- | --- | --- |
| #6 | #5 AND 'Article'/it | 3535 | 7-July-26 |
| #5 | (#1 OR #2) AND (#3 OR #4) | 8331 | 7-July-26 |
| #4 | 'stroke'/exp | 562272 | 7-July-26 |
| #3 | 'acute cerebrovascular accident*':ti,ab,kw OR 'acute stroke*':ti,ab,kw OR 'apoplexy':ti,ab,kw OR 'brain vascular accident*':ti,ab,kw OR 'cerebral stroke*':ti,ab,kw OR 'cerebrovascular accident*':ti,ab,kw OR 'cerebrovascular apoplexy':ti,ab,kw OR 'cerebrovascular stroke*':ti,ab,kw OR 'cva* cerebrovascular accident':ti,ab,kw OR 'stroke*':ti,ab,kw | 665725 | 7-July-26 |
| #2 | 'serotonin uptake inhibitor'/exp | 413636 | 7-July-26 |
| #1 | '5 ht uptake inhibitor*':ti,ab,kw OR '5 hydroxytryptamine uptake inhibitor*':ti,ab,kw OR 'antidepressants, serotonin specific reuptake inhibitors':ti,ab,kw OR 'selective serotonin reuptake inhibitor*':ti,ab,kw OR 'serotonin reuptake inhibitor*':ti,ab,kw OR 'serotonin specific reuptake inhibitor*':ti,ab,kw OR 'serotonin uptake inhibitor*':ti,ab,kw OR 'ssri':ti,ab,kw OR 'ssri antidepressant':ti,ab,kw | 33305 | 7-July-26 |

**Supplementary Table S2.** Main results of included studies. (A) Bleeding event (B) Death event (C) Thrombotic event (D) Cardiac adverse event (E) mRS (F) Recurrent stroke (G) absNIHSS.

**(A)**

| study | n.e | total.e | n.c | total.c |  |
| --- | --- | --- | --- | --- | --- |
| Dennis2019 | 41 | 1564 | 38 | 1563 | 6months |
| Hankey2020 | 4 | 642 | 2 | 638 | 6months |
| Kraglund2018 | 26 | 319 | 20 | 323 | 6months |
| [Lundström2020](https://pubmed.ncbi.nlm.nih.gov/?term=Lundstr%C3%B6m+E) | 10 | 750 | 8 | 750 | 6months |
| Robinson2008 | 1 | 59 | 1 | 58 | 12months |

**(B)**

| study | n.e | total.e | n.c | total.c |  |
| --- | --- | --- | --- | --- | --- |
| He2018 | 4 | 202 | 1 | 202 | (3months) |
| Kraglund2018 | 16 | 319 | 12 | 323 | (6months) |
| Lundström2021 | 34 | 750 | 34 | 750 | (12months) |
| Lundström2021 | 25 | 750 | 22 | 750 | (6months) |
| Oskouie2017 | 4 | 62 | 11 | 61 | (3months) |

**(C)**

| study | n.e | total.e | n.c | total.c |  |
| --- | --- | --- | --- | --- | --- |
| Dennis2019 | 78 | 1564 | 92 | 1563 | 6months |
| Hankey2020 | 12 | 642 | 23 | 638 | 6months |
| Lundström2020 | 8 | 750 | 12 | 750 | 6months |

**(D)**

| study | n.e | total.e | n.c | total.c |  |
| --- | --- | --- | --- | --- | --- |
| Dennis2019 | 15 | 1564 | 23 | 1563 | (6months) |
| Hankey(12months)2021 | 3 | 642 | 3 | 638 | (12months) |
| Hankey(6months)2020 | 1 | 642 | 2 | 638 | (6months) |
| Kraglund2018 | 21 | 319 | 18 | 323 | (6months) |
| [Lundström2020](https://pubmed.ncbi.nlm.nih.gov/?term=Lundstr%C3%B6m+E) | 5 | 750 | 6 | 750 | (6months) |
| Oskouie2017 | 3 | 72 | 8 | 72 | (3months) |
| Robinson2008 | 38 | 59 | 36 | 62 | (12months) |

**(E)**

| study | n.e(0-2 score, achieve favorable scores) | total.e | n.c (0-2 score, achieve favorable scores) | total.c |  |
| --- | --- | --- | --- | --- | --- |
| Bembenek2020 | 20 | 30 | 19 | 31 | 12months |
| Dennis2019 | 572 | 1553 | 588 | 1553 | 6months |
| Hankey(12months)2021 | 446 | 620 | 466 | 630 | 12months |
| Hankey(6months)2020 | 432 | 624 | 458 | 632 | 6months |
| Kraglund2018 | 231 | 319 | 261 | 323 | 6months |
| Lundström2021 | 459 | 715 | 467 | 712 | 12months |
| [Lundström2020](https://pubmed.ncbi.nlm.nih.gov/?term=Lundstr%C3%B6m+E) | 466 | 737 | 475 | 742 | 6months |
| Oskouie(1month)2017 | 34 | 62 | 17 | 61 | 1month |
| Oskouie(3months)2017 | 55 | 62 | 22 | 61 | 3months |

**(F)**

| study | n.e | Ischemic stroke | Haemorrhagic stroke | Intracerebral haemorrhage | Subarachnoid haemorrhage | total.e | n.c | Ischaemic stroke | Haemorrhagic stroke | Intracerebral haemorrhage | Subarachnoid haemorrhage | total.c |  |
| --- | --- | --- | --- | --- | --- | --- | --- | --- | --- | --- | --- | --- | --- |
| Dennis2019 | 56 | 43 | 7 | NA | NA | 1564 | 64 | 45 | 9 | NA | NA | 1563 | 6months |
| Hankey(12months)2021 | 25 | 14 | 6 | NA | NA | 642 | 36 | 29 | 2 | NA | NA | 638 | 12months |
| Hankey(6months)2020 | 18 | 11 | 3 | NA | NA | 642 | 26 | 21 | 1 | NA | NA | 638 | 6months |
| He(36months)2018 | 30 | 30 | NA | NA | NA | 202 | 49 | 49 | NA | NA | NA | 202 | 36months |
| He(3months)2018 | 39 | 39 | NA | NA | NA | 202 | 25 | 25 | NA | NA | NA | 202 | 3months |
| Kraglund2018 | 19 | 19 | NA | NA | NA | 319 | 18 | 18 | NA | NA | NA | 323 | 6months |
| [Lundström2020](https://pubmed.ncbi.nlm.nih.gov/?term=Lundstr%C3%B6m+E) | 36 | 31 | 5 | 5 | 0 | 750 | 28 | 26 | 2 | 1 | 1 | 750 | 6months |
| Oskouie2017 | 4 | 2 | 2 | 2 | NA | 72 | 4 | 1 | 3 | 3 | NA | 72 | 3months |

**(G)**

| study | mean.e | sd.e | n.e | mean.c | sd.c | n.c |  |
| --- | --- | --- | --- | --- | --- | --- | --- |
| Bembenek(12months)2020 | 4.15 | 2.9 | 30 | 5 | 2.84 | 31 | 12months |
| Bembenek(6months)2020 | 3.15 | 1.01 | 30 | 4.71 | 0.85 | 31 | 6months |
| He(0.5month)2016 | 3.02 | 1.97 | 177 | 2.71 | 1.83 | 170 | 0.5month |
| He(3months)2016 | 5.5 | 2.61 | 177 | 4.91 | 2.54 | 170 | 3months |
| He(6months)2016 | 6.15 | 2.97 | 177 | 5.36 | 2.98 | 170 | 6months |
| Lundström2020 | 2.7 | 2.57 | 750 | 2.7 | 2.57 | 750 | 6months |
| Oskouie(1month)2017 | 5.5 | 2.69 | 72 | 5.15 | 3.02 | 72 | 1month |
| Oskouie(3months)2017 | 7.78 | 2.51 | 72 | 7.27 | 2.92 | 72 | 3months |

**Supplementary data S1.** Definition of SSRIs.

Different sources have different definitions and lists of SSRIs. For the purposes of our systematic review, we defined SSRIs as: Citalopram, Paroxetine, Sertraline, Escitalopram, Fluoxetine, Fluvoxamine, Vilazodone, Vortioxetine, Zimelidine, Alaproclate, Etoperidone, Venlafaxine, Desvenlafaxine, Duloxetine, or Milnacipran.
